# Pre-implantation teriparatide administration improves initial implant stability and accelerates the osseointegration process in osteoporotic rats

**DOI:** 10.1186/s40729-024-00536-z

**Published:** 2024-04-16

**Authors:** Boyu Cui, Tianyi Bai, Qiyou Wu, Yibo Hu, Yihong Liu

**Affiliations:** Department of General Dentistry, Peking University School and Hospital of Stomatology, National Center for Stomatology, National Clinical Research Center for Oral Diseases, National Engineering Research Center of Oral Biomaterials and Digital Medical Devices, Central Laboratory, Peking University School and Hospital of Stomatology, Beijing Key Laboratory of Digital Stomatology, NHC Key Laboratory of Digital Stomatology, NMPA Key Laboratory for Dental Materials, Beijing, 100081 China

**Keywords:** Teriparatide, Dental Implants, Osseointegration, Osteoporosis

## Abstract

**Purpose:**

Osteoporotic individuals who have dental implants usually require a prolonged healing time for osseointegration due to the shortage of bone mass and the lack of initial stability. Although studies have shown that intermittent teriparatide administration can promote osseointegration, there is little data to support the idea that pre-implantation administration is necessary and beneficial.

**Methods:**

Sixty-four titanium implants were placed in the bilateral proximal tibial metaphysis in 32 female SD rats. Bilateral ovariectomy (OVX) was used to induce osteoporosis. Four major groups (*n* = 8) were created: PRE (OVX + pre-implantation teriparatide administration), POST (OVX + post-implantation administration), OP (OVX + normal saline (NS)) and SHAM (sham rats + NS). Half of rats (*n* = 4) in each group were euthanized respectively at 4 weeks or 8 weeks after implantation surgery, and four major groups were divided into eight subgroups (PRE4 to SHAM8). Tibiae were collected for micro-CT morphometry, biomechanical test and undecalcified sections analysis.

**Results:**

Compared to OP group, rats in PRE and SHAM groups had a higher value of insertion torque (*p* < 0.05). The micro-CT analysis, biomechanical test, and histological data showed that peri-implant trabecular growth, implants fixation and bone-implant contact (BIC) were increased after 4 or 8 weeks of teriparatide treatment (*p *< 0.05). There was no statistically difference in those parameters between PRE4 and POST8 subgroups (*p* > 0.05).

**Conclusions:**

In osteoporotic rats, post-implantation administration of teriparatide enhanced peri-implant bone formation and this effect was stronger as the medicine was taken longer. Pre-implantation teriparatide treatment improved primary implant stability and accelerated the osseointegration process.

## Introduction

Osteoporosis is a disease which can lead to a decrease in bone density and bone mass. Osteoporosis-induced loss of bone mass and deterioration of bone microarchitecture can also impact primary implant stability and the biological process of peri-implant bone regeneration in both human and animal models [1]. According to Pan et al.‘s findings, osteoporotic rats had insufficient bone volume surrounding implants and a lower value of bone-implant contact (BIC) [2]. And consequently, extending the healing period is an essential clinical approach to attain the requisite osseointegration in patients suffering from osteoporosis [3].

Teriparatide, a recombinant human parathyroid hormone (PTH) composed of the hormone’s 1–34 amino acids, has been approved by the Food and Drug Administration (FDA) for the treatment of adults with severe osteoporosis. Its anabolic effect has been demonstrated to promote bone formation [4, 5]. Small doses of intermittent administration of teriparatide also have a positive impact on implants osseointegration. After administered teriparatide for 12 weeks, new bone formation was found around external and internal surfaces of titanium implants in osteoporotic rabbits [6]. And following teriparatide treatment, osteoporotic SD rats also showed an increased value of maximal pull-out force as a sign of increased implant fixation [7]. Similarly, Li et al.‘s investigation further showed how teriparatide treatment altered the peri-implant bone mass and density associated to an increase in implant fixation in osteoporotic rats by micro-CT and histological results [8].

Kuchler et al. [9] conducted a clinic feasibility study involving 24 participants, the median age of whom was 63. After nine weeks, the implants—which included “study implants”—were removed using a trephine from the edentulous lower jaws. They found that the new bone volume per tissue volume (NBV/TV) and new bone-implant contact (NBIC) increased, which was in line with the outcomes of animal experiments and offered the first histology data on the osseointegration of titanium implants in patients treated with teriparatide.

In earlier pre-clinical studies, teriparatide was administered for three to four weeks prior to implantation and continued until the animal was euthanized [10–12]. Their findings suggested improved biomechanical strength at the implant-bone interface. Nevertheless, in contrast to studies where teriparatide was provided post-implantation [6–8, 13], there was scant evidence to bolster the notion that pre-implantation care could confer greater advantages. Furthermore, there are relatively few clinical reports of teriparatide treatment prior to implantation in patients with osteoporosis, despite the fact that implants in these patients typically lack primary stability due to low local bone densities [1].

The aim of this paper is to prove the positive effect of pre-implantation administration of teriparatide in osteoporotic rats. We hypothesize that teriparatide treatment prior to implantation surgery may improve the initial implant stability and accelerate the osseointegration process.

## Materials and methods

### Study design

The animal experiments in this study were approved by the Peking University Institutional Review Board (PUIRB) with the registration number (PUIRB-LA2023295). Female SD rats (Vitalriver, Beijing, China) aged 8 to 9 months were housed in controlled environmental conditions, with a temperature of 25 °C, humidity of 55%, and a 12-hour light-dark cycle.

**Fig. 1:**
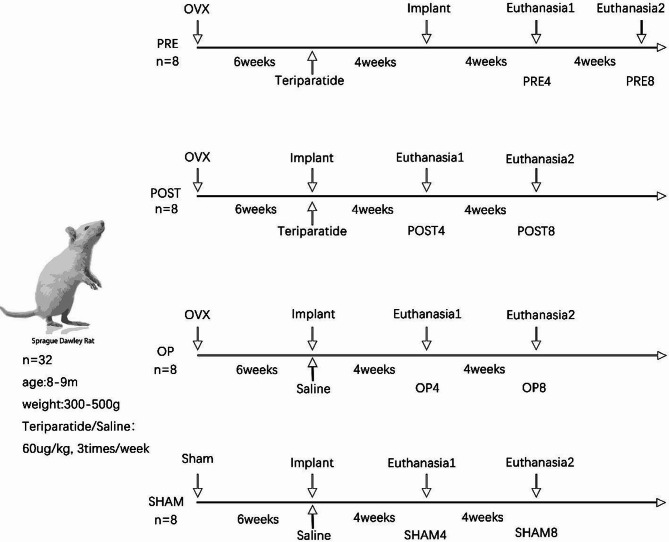
Experimental design is shown above. A total of 32 female SD rats (8–9 months old) were divided into 4 major groups: PRE, POST, OP and SHAM. Titanium implants were placed in the bilateral proximal tibiae. Rats in each group (*n* = 8) were euthanized at 4 weeks (*n* = 4) or 8 weeks (*n* = 4) after implantation and the four major groups were divided into eight subgroups (PRE4, PRE8, POST4, POST8, OP4, OP8, SHAM4 and SHAM8). After euthanasia, 8 tibiae were collected in each subgroup

As shown in Fig. 1, a total of 32 rats were randomly divided into 4 major groups: PRE, POST, OP and SHAM. After bilateral ovariectomy (OVX) or sham surgery, rats were fed with maintain feed which containing 1.4% Ca, 0.8% P and water for 42 days to induce osteoporosis according to a previous research [14]. In PRE group, the administration of teriparatide (XINFUTAI, Shenzhen, Guangdong, China) commenced at 4 weeks prior to the implantation. In POST group, the administration started with the implantation. As a blank control group, rats in OP group received an injection of normal saline (NS) after implantation. And in SHAM group, the implants were placed 42 days after undergoing a sham surgery and then NS was injected. Half of rats were euthanized at 4 or 8 weeks after implantation surgery and four major groups were divided into eight subgroups (*n* = 4). Rats in PRE4-SHAM4 subgroups were euthanized at 4 weeks, and rats in PRE8-SHAM8 subgroups were euthanized at 8 weeks.

Previous research suggests that daily PTH treatment may raise the concentration of the bone resorption marker [15], whereas teriparatide administered three times a week promotes bone formation on the endocortical surface without improving bone turnover markers, resulting in an increase in cortical thickness and bone density [16]. Consequently, we administered 60 µg/kg of teriparatide or NS subcutaneously three times a week, which might promote implant osseointegration effectively.

### Validation of osteoporosis

The serum concentration of osteocalcin (OCN) and type I collagen C-terminal telopeptide (CTX-I) were measured to verify the successful establishment of osteoporosis models [17, 18]. Blood samples were randomly collected from five osteoporosis rats (in PRE, POST and OP groups) and five normal rats (in SHAM group), and centrifuged at 1000 x g for 20 min using a high-speed freeze centrifuge (Thermo Fisher, Waltham, MA, USA). The serum concentrations of OCN and CTX-I was tested using the OCN Elisa serum test kit (Sabbiotech, College Park, MD, USA) and the CTX-I Elisa protein test kit (Cloud-Clone, Wuhan, Hubei, China) separately. The absorption intensity of each well at 450 nm was analyzed using a multimode microplate detector (PerkinElmer, Waltham, MA, USA) to establish a standard sample function curve, and the concentrations of OCN and CTX-I were calculated based on this curve.

### Implantation procedure

Sixty-four grade IV pure titanium screws (BioMaterials, Beijing, China), with 2 mm in diameter and 5 mm in length, were chosen to simulate the dental implants. The self-tapping implant had four continuous threads, and the special design of the tip facilitated the removal of bone debris during the implantation process.

The abdominal injection of Zoletil@50 (Aladdin, Shanghai, China) was used for anesthesia with the dosage of 307 mg/kg. After anesthesia, the skin was incised at the proximal tibia to expose the underlying tibial surface, and each rat then received an implant in the right and left tibial metaphysis respectively. Using a bone drill (DISHENGDE, Jinhua, Zhejiang, China), we bored a pilot hole 2–3 mm away from the tibial growth plate and perpendicular to the medial surface of tibial metaphyseal under NS flow. The hole measured 1 mm in diameter and 3 mm in length. Then with the help of a torque screwdriver (AIGU, Dongguan, Guangdong, China), the implant was progressively inserted in the pilot hole until the point where every thread was fully inside the bone. The maximal torque encountered in PRE, OP and SHAM groups were recorded during the insertion process, indicating the insertion torque. Finally, the skin and mucous membranes were sutured to close the incision site.

The animals in PRE and POST groups were continually treated with teriparatide after implant operation, while rats in OP and SHAM groups were injected NS. Rats were euthanized by excessive CO_2_ inhalation following euthanasia principles. Eight tibiae from four rats in each subgroup were collected 4 or 8 weeks after implantation. Then the specimens were fixed in a 10% formalin neutrophilic fixation (Yulu, Nanchang, Jiangxi, China) for one week. Three tibiae were randomly collected from different rats preparing for undecalcified sections analysis, while the rest five tibiae were subjected to micro-CT scanning and torque test.

### Micro-CT analysis

After fixation, 5 tibiae were scanned by micro-CT (Bruker Skyscan 1172, Aatselaar, Antuérpia, Belgium), with parameters included a voltage of 90 kV, a current of 80 µA, an exposure time of 1050 ms, a rotation angle of 180°, a rotational step length of 0.4°, a filter of 0.5 mm AL, and a scan resolution of 13.75 μm. Then the images were reconstructed three dimensionally and analyzed using the Data Viewer software (SkyScan, version 1.5.6.2) and the CTAn software (SkyScan, version 1.18.8.0).

**Fig. 2:**
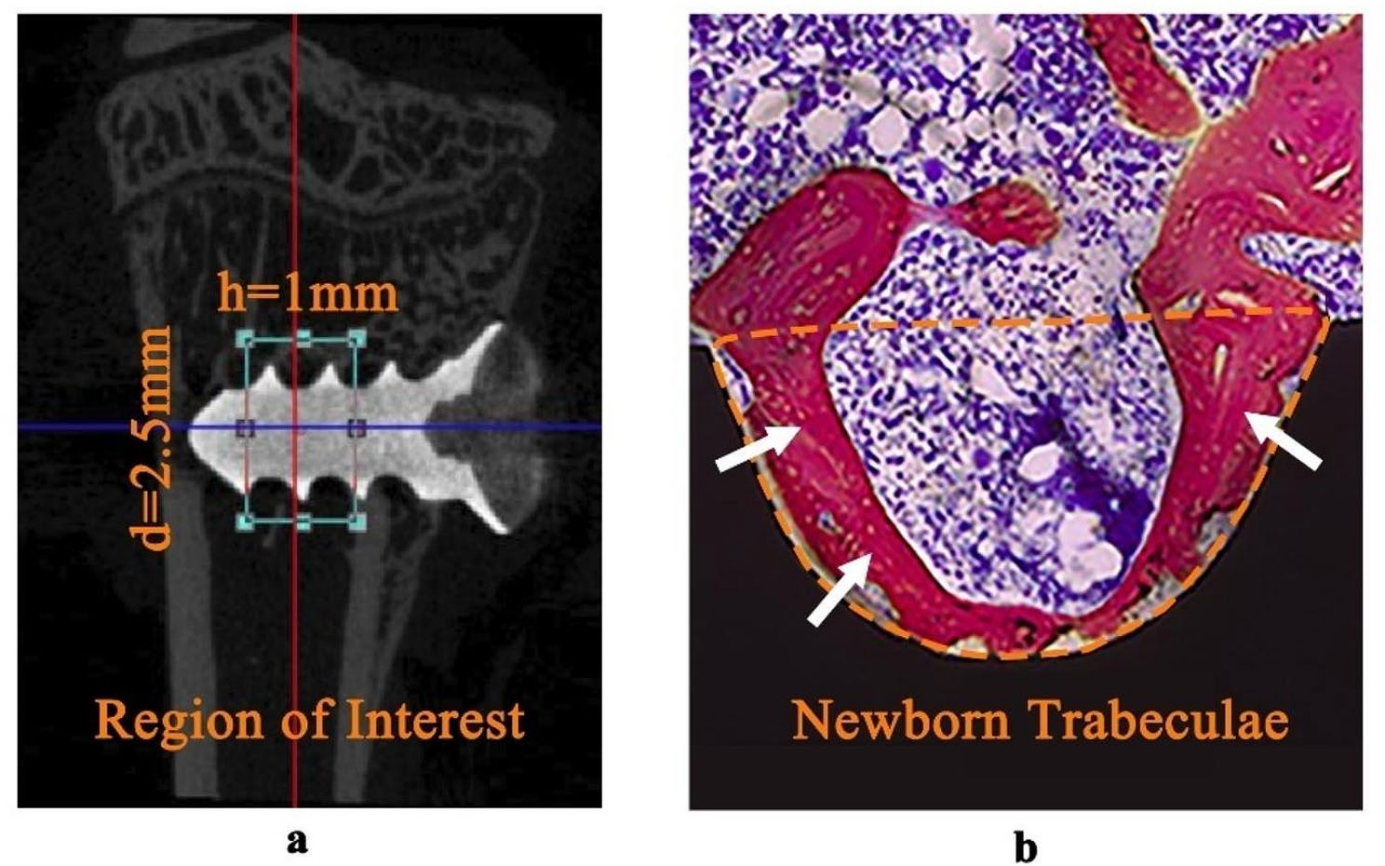
(**a**) The area surrounding the implant, measuring 2.5 mm in diameter and 1 mm in height, was designated as the region of interest. (**b**) The trabeculae within the orange dashed line were identified as newborn trabeculae in thread pits (marked by white arrows). Thread’s BIC was defined as the square of the newborn trabeculae in the thread pit divided by the overall square of the thread pit

The Region of interest (ROI) was defined as the area around implants and the bone tissues (Fig. 2a, **2**.5 mm in diameter and 1 mm in height). The CTAn software was used to quantify various metrics within the ROI, including bone volume percentage (BV/TV), trabecular thickness (Tb.Th), trabecular number (Tb.N) and trabecular separation (Tb.Sp). For the CTAn analysis, a grayscale option ranging from 40 to 80 was selected.

### Biomechanical testing

Immediately after micro-CT scanning, the five tibiae with implants in each subgroup were fixed by a torque horizontal clamp and connected to a digital torque meter (HBO, Beijing, China), in anticipation of the torque test. A screwdriver was used to remove the implant from the bone and the maximal removal torque needed to remove the implant was recorded during this process.

### Undecalcificated bone-implant sections

After fixation, the three tibiae underwent a dehydration process using a graded series of ethanol concentrations: 70%, 80%, 90%, and finally 100%. Following dehydration, the tibiae were soaked in a solution consisting of 50% ethanol and 50% light-curing resin (Technovit 7200VLC, Hanau, Germany) for a period of 2 weeks. Subsequently, the tibiae were embedded in resin (Technovit 7200VLC, Hanau, Germany) and photopolymerized for 10 h. A special fixed glue (Tech 4000, Hanau, Germany) was used to fix the specimens block to the cover glass, and the EXAKT hard tissue cutting system (EXAKT 300CP, Apparatebau, Gmbh, Hamburg, Germany) was used to cut vertically along the long axis of the implant at a thickness of 100 μm, and grinded in the polishing machine (EXAKT 400CS, Apparatebau, Gmbh, Hamburg, Germany). Then the hard tissue sections were dyed using methylene-blue/acid fuchsin stain to visualize trabecular bone growth around the implants. Finally, the multi-mode intelligent living cells imaging analysis system (MICA) (Leica, Wetzlar, Hessian, Germany) was used to observe the growth patterns of trabeculae. Thread’s BIC was defined as the square of the newborn trabeculae in the thread pit (Fig. 2b) divided by the overall square of the thread pit. And the overall BIC of the section was defined as the average of the two consecutive threads’ BIC values in the cancellous bone area. After locally enlarged images of two consecutive threads (in cancellous bone area) were transported to software ImageJ (Image Processing and Analysis Software, Bethesda, MD, USA), the values of overall BIC of different subgroups were calculated and compared.

### Statistical analysis

The SPSS 24.0 software (SPSS, Chicago, IL, USA) was used throughout for statistical analysis. One-way analysis of variance (one-way ANOVA) was used to compare the radiological, biomechanical and histological parameters among groups or subgroups. When ANOVA indicated significant differences, post-hoc multiple comparisons were conducted using either the Bonferroni method (in cases of homogeneity of variance) or the Tamheini test (in cases of heterogeneity of variance). To evaluate differences in serum concentrations of OCN and CTX-I between osteoporotic and sham rats, and to analyze the difference of parameters in PRE4 and POST8, a two-independent sample t-test was employed. A significance level of *p* < 0.05 was used to determine statistical significance. All values are shown as the average and the standard difference.

## Results

### Confirmation of osteoporosis model

The average serum OCN concentration of osteoporotic rat was 29.59 ± 6.70 ng/ml, while sham rat was 17.43 ± 3.3 ng/ml, with statistically significant difference (*P* = 0.007). Similarly, the serum CTX-I concentration of osteoporotic rats (449.20 ± 72.84 pg/ml) was significantly higher than that of sham rats (139.23 ± 15.68 pg/ml). The obviously increased serum concentration of OCN and CTX-I in osteoporotic rats, as markers of bone metabolism, indicated that the models of osteoporosis were successfully established at 42 days after bilateral ovariectomy.

### Micro-CT analysis

**Fig. 3:**
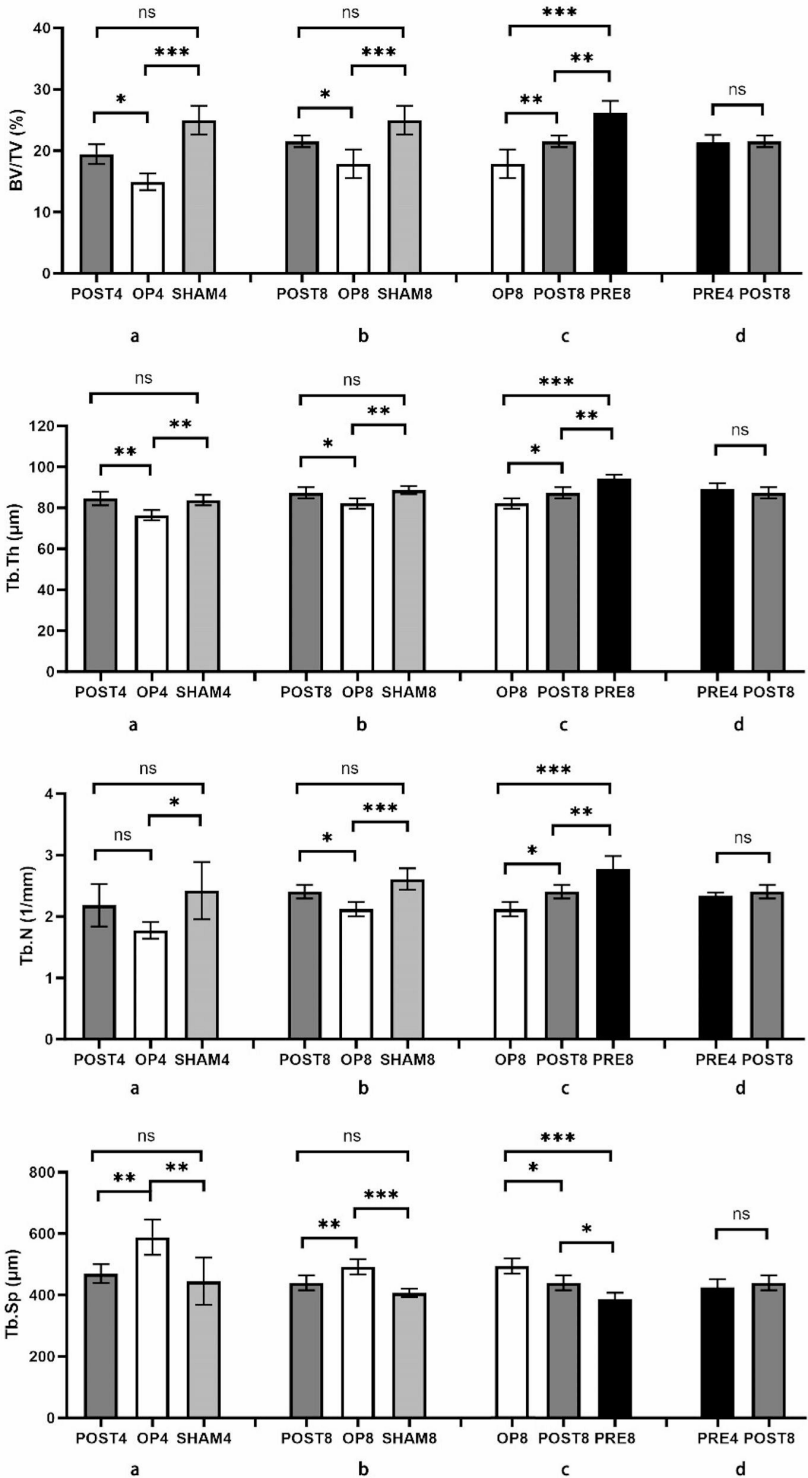
Radiology parameters of BV/TV, Tb.Th, Tb.N, Tb.Sp were analyzed statistically. Values expressed are means ± SD, *n* = 5, *p < 0.05, **p < 0.01, ***p < 0.001, significantly different compared between two subgroups. “ns”, no significant difference. (**a**) Rats in POST4, OP4 and SHAM4 were compared. (**b**) Rats in POST8, OP8 and SHAM8 were compared. (**c**) Rats in OP8, POST8 and PRE8 were compared to evaluate the effect of teriparatide administration with different durations (0 week, 8weeks, 12 weeks). (**d**) There was no significant difference of radiological parameters between PRE4 and POST8 (*p* > 0.05)

Through micro-CT scanning and analysis, the radiology parameters of BV/TV, Tb.Th, Tb.N, Tb.Sp were evaluated. The results were shown in Fig. 3.

Rats in POST, OP and SHAM groups were compared to estimate the effect of teriparatide administration after implantation. As revealed in Fig. 3a, there was no considerable difference in BV/TV, Tb.Th, Tb.Sp, and Tb.N when comparing POST4 and SHAM4 (*p* > 0.05). Similarly, as shown in Fig. 3b, no significant difference was found in those radiology parameters in POST8 and SHAM8 (*p* > 0.05). And when compared to OP group, the values of BV/TV and Tb.Th were obviously enhanced and the value of Tb.Sp was pronouncedly decreased in POST and SHAM groups (Fig. 3a and b). These results revealed that the peri-implant trabecular bone grew and returned to the normal level after 4- or 8-week-course of teriparatide treatment.

Furthermore, rats in OP8, POST8, and PRE8 were compared to estimate if the different duration of teriparatide administration could make a difference to trabecular bone growth. The only difference among the rats in OP8, POST8, and PRE8—who were all osteoporotic and euthanized at 8 weeks after implantation—was how long their medicine was given (0 week, 8 weeks and 12 weeks respectively). When OP8 was compared to PRE8 and POST8, values of BV/TV, Tb.Th, Tb.N, and Tb.Sp showed statistically significant differences (Fig. 3c, *p* < 0.05). Additionally, there was a significant difference between POST8 and PRE8 in terms of those radiological parameters (*p* < 0.05), which demonstrated an increased trend of trabecular bone growth as the administration time extended.

Finally, we compared rats in PRE4 and POST8 to reveal the impact of teriparatide administration before implantation. Rats in subgroups PRE4 and POST8 received the same dose and duration (8 weeks) of teriparatide administration. Rats in PRE4 received medicine for four weeks prior to and after implantation, whereas rats in POST8 got treatment after implantation. As a result, rats in PRE4 had a shorter bone healing period (4 weeks) than those in POST8 (8 weeks). According to the statistic results (Fig. 3d), no considerable differences were found between them in BV/TV, Tb.Th, Tb.N and Tb.Sp (*p* > 0.05). It indicated that the peri-implant bone volume and trabecular bone growth in PRE4 were consistent with those in POST8, though the bone healing time of rats in PRE4 was less than those in POST8.

### Biomechanical testing

#### Maximal insertion torque

**Fig. 4:**
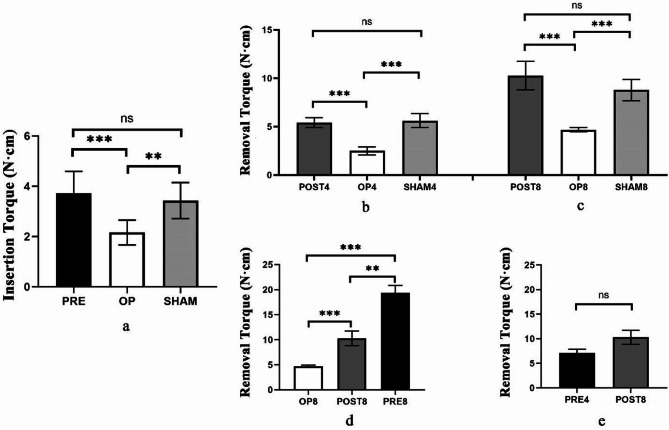
Biomechanical results were expressed as the maximal insertion torque and removal torque. Values expressed are means ± SD, *n* = 5, *p < 0.05, **p < 0.01, ***p < 0.001, significantly different compared between two groups or subgroups. “ns”, no significant difference. (**a**) The results of maximal insertion torque in PRE, OP and SHAM groups. (**b**) & (**c**) The comparison of maximal removal torque in groups POST, OP and SHAM. (**d**) Comparison of maximal removal torque in OP8, POST8 and PRE8. (**e**) Maximal removal torque was compared in PRE4 and POST8

In Fig. 4a, the maximal insertion torque values for rats in groups PRE, OP and SHAM were 3.72 (± 0.87) N·cm, 2.16 (± 0.49) N·cm, and 3.43 (± 0.72) N·cm, respectively. Comparing rats in PRE and SHAM groups, no statistically significant differences were found (*p* > 0.05). And the maximal insertion torque values were higher in PRE and SHAM groups compared to OP group (*p* < 0.05).

#### Maximal removal torque

According to Fig. 4b and c, the maximal removal torque of rats in POST, OP and SHAM groups were compared together to exam the effect of teriparatide on implants fixation. No statistically significant difference was found between POST4 and SHAM4, as well as between POST8 and SHAM8 (*p* > 0.05). And when compared to OP group, the torque test demonstrated increased maximal removal torque in both POST and SHAM groups (*p* < 0.05), which implied that teriparatide could promote implants fixation after administering for 4 or 8 weeks in POST group.

Besides, when comparing the maximal removal torque in PRE8 (19.32 ± 1.53 N·cm), POST8 (10.28 ± 1.47 N·cm) and OP8 (4.68 ± 0.22 N·cm) (Fig. 4d), significant differences were found among them (*p* < 0.05). And rats in PRE8 had the highest maximal removal torque, correlated with the longest duration of teriparatide administration.

Furthermore, it was surprised to find the maximal removal torque values in PRE4 and POST8 had not shown any significant differences (*p* > 0.05), despite the fact that bone healing time of rats in POST8 was double that of rats in PRE4 (Fig. 4e). Therefore, it could be concluded that the pre-implantation teriparatide administration promoted implants fixation and accelerated the osseointegration process.

### Histological evaluation

**Fig. 5:**
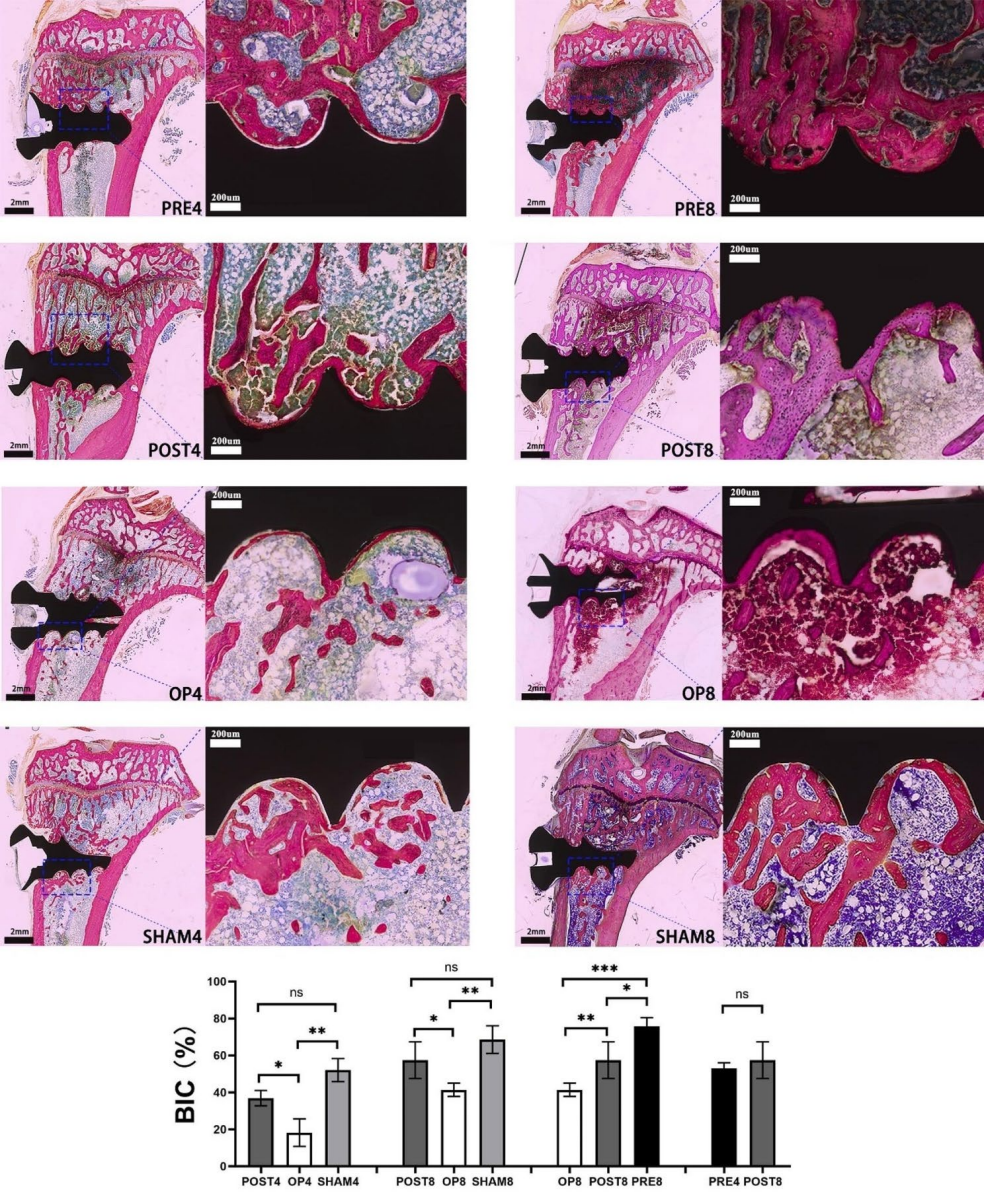
Representative methylene blue/acid fuchsin-stained histological section of the bone around titanium implants were examined by MICA system. Red color indicates trabecular bone and cortical bone, while blue color represents the collagen and bone cells. Locally enlargement (magnification ×500) pictures of the two consecutive threads were transported to ImageJ to calculate the overall BIC values. Difference of those results among subgroups were compared. Values expressed are means ± SD, *n* = 3, *p < 0.05, **p < 0.01, ***p < 0.001, significantly different compared between two subgroups. “ns”, no significant difference

To investigate the peri-implant bone formation and the contact of tibiae and implants, tibial specimens were stained in methylene blue/acid fuchsin. While the trabecular or cortical bone were dyed bright pink, the collagen or bone cell were dyed light blue (Fig. 5).

Rats in SHAM group served as a representation of the normal rats, with bone-implant histology appearance reflecting typical osseointegration phases. Even though there was still gaps between the implant and the tibia after eight weeks of osseointegration, peri-implant bone formation in SHAM8 was more evident than that in SHAM4. Additionally, only scattered trabecular bone were detected around the implants in OP group, which had the fewest newborn trabeculae both in terms of number and thickness. Although there were thicker trabeculae in OP8 when comparing to those in OP4, the bone density of metaphyseal tibia in OP8 was still low and aligned well with typical histological findings of osteoporosis. The PRE and POST groups exhibited increased trabecular number and decreased bone cells or fibrous tissues after teriparatide administration, and the values of trabecular thickness in PRE and POST groups had been almost similar with or even higher than those in SHAM group. In PRE8, the highest level of peri-implant bone growth was detected as the newly formed trabeculae were continuous and firmly adhered to the implant’s threads. And the thickness and quantity of trabeculae were almost similar with each other when comparing rats’ specimens between PRE4 and POST8.

In order to study the values of overall BIC in different subgroups, we magnified (×500) the area of two consecutive threads in cancellous bone area. According to the results in Fig. 5, there were no statistically difference in overall BIC values when POST and SHAM groups were compared (POST4 vs. SHAM4 or POST8 vs. SHAM8, *p* > 0.05). Overall BIC values were much higher in POST4 compared to OP4, and there was also a statistically significant difference in those values between POST8 and OP8 (*p* < 0.05), which indicated the impact of teriparatide therapy. Additionally, a longer duration of teriparatide administration improved its effects, as seen by significant differences of overall BIC values among OP8, POST8, and PRE8 (*p* < 0.05). In the end, there was no discernible change in those values between PRE4 and POST8 (*p* > 0.05), indicating that despite the differences in the bone healing periods, the contact of bone and implants was similar following the same duration of teriparatide administration. In other words, administering teriparatide for four weeks prior to implantation reduced the amount of time needed for osseointegration.

## Discussion

The most significance of this study is to evaluate the impact of pre-implantation administration of teriparatide in osteoporotic rats. So far, few research has emphasized the necessity of medication prior to implantation. Additionally, it is the first to evaluate the variations in implant osseointegration between pre- and post-implantation teriparatide treatment.

Initial stability is a prerequisite for achieving osseointegration of implants [19]. However, patients with osteoporosis typically experience a higher failure rate due to the lack of initial stability [1, 20]. Various methods can be used to measure the initial stability, including X-ray analysis, resonance frequency analysis (RFA), and insertion torque. While studies have shown a correlation between the initial implant stability quotient (ISQ) value and the maximal insertion torque [21, 22], it’s crucial to note that an extension rod is needed to be connected to the implant once the ISQ value is calculated. However, the desired implant’s diameter is a little too large, making it inappropriate to be implanted in rats’ tibiae. Consequently, we chose the maximal insertion torque as the measurement of initial implants stability. Based on the results of insertion torque from PRE, OP and SHAM groups, the early subcutaneous injection of teriparatide for four weeks significantly increased the maximal insertion torque (*p* < 0.05). And this suggested that the pre-implantation administration of teriparatide could effectively enhance the initial implants stability in osteoporotic rats. Given these circumstances, administering teriparatide prior to implantation surgery to improve initial implants stability might be an option for those with osteoporosis who need dental implants restoration.

The efficacy of post-implantation administration of teriparatide in promoting peri-implant bone growth and implant fixation was confirmed by the results of improved trabecular quality or quantity in osteoporotic rats and increased maximal removal torque. In osteoporotic rats, the post-implantation administration of teriparatide may stimulate the growth of peri-implant bone after four or eight weeks. As a result, as seen by the outcomes of the POST groups, implant fixation was clearly improved, and implant osseointegration was seen to be greater both radiologically and histologically when compared with the OP group. Our results align well with previous researches. Li et al. [23] exhibited the formation of new bone surrounding implants in ovariectomized rats by administering teriparatide and menaquinone-4 (vitamin K2; MK). Additionally, Li et al. [8] illustrated the combined benefits of teriparatide and zoledronic acid on implant fixation in ovariectomized rats. Their dosages were the same as ours—60 µg/kg, three times a week. Furthermore, our research indicated that, following teriparatide treatment for 4 or 8 weeks, the peri-implant bone promoting effect in osteoporotic rats can be restored to the same degree of normal rats, as seen by the outcomes in the POST and SHAM groups.

To evaluate the impact on osseointegration between pre-implantation and post-implantation teriparatide administration, the comparison of rats in PRE4 and POST8 was conducted. Both rats in PRE4 and POST8 were given teriparatide for 8 weeks, and there were two healing phases (time for osseointegration) of 4 weeks and 8 weeks. Surprisingly, in terms of results of BV/TV, Tb.Th, Tb.N, Tb.Sp, maximal removal torque and the overall BIC results, there were no statistically significant differences of those parameters in PRE4 when compared to POST8 (*p* > 0.05). This suggested that the implants in both subgroups had similar levels of osseointegration, and it also revealed that administering teriparatide for four weeks prior to implant surgery could speed up the osseointegration process. Based on the limited results in this study, we deduced the possibility to accelerate the process of osseointegration by pre-implantation administration of teriparatide in osteoporotic patients.

As is well known, there are multiple stages to the healing process that lead to osseointegration in healthy patients: growth factors are produced, osteoblasts migrate, and neutrophils and macrophages are recruited on days 1 and 2 after implantation [24–26]. Necrotic bone resorption and new bone deposition at the implant-bone contact occur in the days that follow. And on day 28, the bone begins to restructure as new layers of bone formed adjacent to the implant [27]. However, in ovariectomized rats, the lack of estrogen may interact with various immune cells, leading to a chronic pro-inflammatory phenotype. Additionally, certain cytokines, such as tumor necrosis factor α (TNFα), have been shown to cause osteoblast apoptosis [28, 29]. Therefore, osteoporotic patients usually need a longer healing period to achieve osseointegration [3].

Teriparatide has a pharmacological mechanism that involves directly activating survival signaling in osteoblasts and delaying osteoblast apoptosis via the cyclic adenosine monophosphate (cAMP)/protein kinase A (PKA) signal transduction pathway, which is a major contributing factor in the increased number of osteoblasts [30]. Furthermore, teriparatide stimulates active osteoclasts and hematopoietic cells differentiation via the nuclear factor-kB (RANK)/RANK Ligand (RANKL)/osteoprotegerin (OPG) pathway [31, 32]. These also happened in the area around the implants: in comparison to the control group, systemic teriparatide injection dramatically enhanced the number of osteocytes, osteoblasts, and osteoclasts inside the implants’ thread area [33]. As previously stated, osseointegration is a physiological process that involves the balance between bone formation and destruction, which is controlled by osteoblasts and osteoclasts. Initial bone matrix absorption by osteoclasts, osteoblast recruitment, and osteoblast adherence to the implant’s surface are all necessary for the subsequent osseointegration of the implant [34, 35]. Pre-implantation teriparatide administration for four weeks in our study boosted osteogenic cells and improved bone metabolism in advance of implantation surgery, which might facilitate cell recruitment, bone remodeling and bone growth in the peri-implant bone areas. As a result, during the process of bone healing, rats’ implants in the PRE group obtained osseointegration faster than those in the POST group.

There were several limitations in this study. Although the contribution of rat models is undeniable, rat bone tissue does not exhibit obvious Haversian remodeling. Osteon-like structures are occasionally seen in rats, but they are not as well-organized as those in bigger animals [36], let alone humans. Even so, rats were still our best choice for inducing osteoporosis because of the large quantity of animals required in our experiment and the ethical requests. Furthermore, it is important to note that the findings of this study are limited to animal experiments and have not been applied in human clinical trials. Therefore, caution should be exercised when extrapolating these results to human patients.

## Conclusions

In osteoporotic rats, post-implantation administration of teriparatide enhanced peri-implant bone formation and this effect was stronger as the medicine was taken longer. Pre-implantation teriparatide treatment improved primary implant stability and accelerated the osseointegration process.

## Data Availability

The datasets used and/or analyzed during the current study are available from the corresponding author on reasonable request.

## Abbreviations

OVX: Ovariectomy
NS: Normal saline
BIC: Bone-implant contact
PTH: Parathyroid hormone
FDA: Food and Drug Administration
NBV/TV: New bone volume per tissue volume
NBIC: new bone-implant contact
OCN: Osteocalcin
CTX-I: Type I collagen C-terminal telopeptide
BV/TV: Bone volume percentage
Tb.Th: Trabecular thickness
Tb.N: Trabecular number
Tb.Sp: Trabecular separation
MICA: Multi-mode intelligent living cells imaging analysis system
ANOVA: Analysis of variance
RFA: Resonance frequency analysis
ISQ: Implant stability quotient
TNFα: Tumor necrosis factor α
vitamin K2; MK: menaquinone-4
cAMP: Cyclic adenosine monophosphate
PKA: Protein kinase A
RANK: Nuclear factor-kB
RANKL: RANK Ligand
OPG: Osteoprotegerin

## References

[1] Merheb J, Temmerman A, Rasmusson L, Kübler A, Thor A, Quirynen M (2016). Influence of skeletal and local bone density on Dental Implant Stability in patients with osteoporosis. Clin Implant Dent Relat Res.

[2] Pan J, Shirota T, Ohno K, Michi K (2000). Effect of ovariectomy on bone remodeling adjacent to hydroxyapatite-coated implants in the tibia of mature rats. J Oral Maxillofac Surg.

[3] Tsolaki IN, Madianos PN, Vrotsos JA (2009). Outcomes of dental implants in osteoporotic patients. A literature review. J Prosthodont.

[4] Vall H, Parmar M. Teriparatide. StatPearls [Internet]. Treasure Island (FL): StatPearls Publishing; 2023 [cited 2023 Dec 15]. http://www.ncbi.nlm.nih.gov/books/NBK559248/.

[5] Harada S, Rodan GA (2003). Control of osteoblast function and regulation of bone mass. Nature.

[6] Almagro MI, Roman-Blas JA, Bellido M, Castañeda S, Cortez R, Herrero-Beaumont G (2013). PTH [1–34] enhances bone response around titanium implants in a rabbit model of osteoporosis. Clin Oral Implants Res.

[7] Zhou C, Wang Y, Meng J, Yao M, Xu H, Wang C (2022). Additive effect of parathyroid hormone and zoledronate acid on Prevention Particle wears-Induced Implant Loosening by promoting Periprosthetic Bone Architecture and Strength in an Ovariectomized Rat Model. Front Endocrinol (Lausanne).

[8] Li YF, Li XD, Bao CY, Chen QM, Zhang H, Hu J (2013). Promotion of peri-implant bone healing by systemically administered parathyroid hormone (1–34) and zoledronic acid adsorbed onto the implant surface. Osteoporos Int.

[9] Kuchler U, Luvizuto ER, Tangl S, Watzek G, Gruber R (2011). Short-term teriparatide delivery and osseointegration: a clinical feasibility study. J Dent Res.

[10] Gomes-Ferreira PHS, Frigério PB, de Moura J, Duarte ND, de Oliveira D, Deering J (2023). Evaluation of vitamin D isolated or Associated with Teriparatide in Peri-implant Bone Repair in Tibia of Orchiectomized rats. Biology (Basel).

[11] Gomes-Ferreira PHS, de Oliveira D, Frigério PB, de Souza Batista FR, Grandfield K, Okamoto R (2020). Teriparatide improves microarchitectural characteristics of peri-implant bone in orchiectomized rats. Osteoporos Int.

[12] Shibamoto A, Ogawa T, Duyck J, Vandamme K, Naert I, Sasaki K (2018). Effect of high-frequency loading and parathyroid hormone administration on peri-implant bone healing and osseointegration. Int J Oral Sci.

[13] Tao Z-S, Zhou W-S, Bai B, Cui W, Lv Y-X, Yu X-B (2016). The effects of combined human parathyroid hormone (1–34) and simvastatin treatment on the interface of hydroxyapatite-coated titanium rods implanted into osteopenic rats femurs. J Mater Sci Mater Med.

[14] Yousefzadeh N, Kashfi K, Jeddi S, Ghasemi A (2020). Ovariectomized rat model of osteoporosis: a practical guide. EXCLI J.

[15] Fox J, Miller MA, Newman MK, Metcalfe AF, Turner CH, Recker RR (2006). Daily treatment of aged ovariectomized rats with human parathyroid hormone (1–84) for 12 months reverses bone loss and enhances trabecular and cortical bone strength. Calcif Tissue Int.

[16] Takao-Kawabata R, Isogai Y, Takakura A, Shimazu Y, Sugimoto E, Nakazono O (2015). Three-times-weekly administration of teriparatide improves vertebral and peripheral bone density, microarchitecture, and mechanical properties without accelerating bone resorption in ovariectomized rats. Calcif Tissue Int.

[17] Hong N, Kim JE, Lee SJ, Kim SH, Rhee Y (2018). Changes in bone mineral density and bone turnover markers during treatment with teriparatide in pregnancy- and lactation-associated osteoporosis. Clin Endocrinol (Oxf).

[18] Eastell R, Szulc P (2017). Use of bone turnover markers in postmenopausal osteoporosis. Lancet Diabetes Endocrinol.

[19] Tabassum A, Meijer GJ, Wolke JGC, Jansen JA (2009). Influence of the surgical technique and surface roughness on the primary stability of an implant in artificial bone with a density equivalent to maxillary bone: a laboratory study. Clin Oral Implants Res.

[20] Tian T, Liu H-H, Zhang Z-H, Han Q, Chen J, Lv J (2022). Correlation between bone volume fraction in posterior implant area and initial implant stability. Oral Surg Oral Med Oral Pathol Oral Radiol.

[21] do Souza V, de Moraes Melo Neto JP, Piacenza CL, Freitas da Silva LT, de Melo Moreno EV, Penitente AL (2021). Relation between insertion Torque and Implant Stability Quotient: a clinical study. Eur J Dent.

[22] Park K-J, Kwon J-Y, Kim S-K, Heo S-J, Koak J-Y, Lee J-H (2012). The relationship between implant stability quotient values and implant insertion variables: a clinical study. J Oral Rehabil.

[23] Li H, Zhou Q, Bai B-L, Weng S-J, Wu Z-Y, Xie Z-J (2018). Effects of combined human parathyroid hormone (1–34) and menaquinone-4 treatment on the interface of hydroxyapatite-coated titanium implants in the femur of osteoporotic rats. J Bone Min Metab.

[24] Cooper LF (1998). Biologic determinants of bone formation for osseointegration: clues for future clinical improvements. J Prosthet Dent.

[25] Rajpurohit R, Koch CJ, Tao Z, Teixeira CM, Shapiro IM (1996). Adaptation of chondrocytes to low oxygen tension: relationship between hypoxia and cellular metabolism. J Cell Physiol.

[26] Depprich R, Zipprich H, Ommerborn M, Mahn E, Lammers L, Handschel J (2008). Osseointegration of zirconia implants: an SEM observation of the bone-implant interface. Head Face Med.

[27] Büchter A, Joos U, Wiesmann H-P, Seper L, Meyer U (2006). Biological and biomechanical evaluation of interface reaction at conical screw-type implants. Head Face Med.

[28] Pacifici R (1996). Estrogen, cytokines, and pathogenesis of postmenopausal osteoporosis. J Bone Min Res.

[29] Ralston SH (1994). Analysis of gene expression in human bone biopsies by polymerase chain reaction: evidence for enhanced cytokine expression in postmenopausal osteoporosis. J Bone Min Res.

[30] Jilka RL (2007). Molecular and cellular mechanisms of the anabolic effect of intermittent PTH. Bone.

[31] Onyia JE, Miles RR, Yang X, Halladay DL, Hale J, Glasebrook A (2000). In vivo demonstration that human parathyroid hormone 1–38 inhibits the expression of osteoprotegerin in bone with the kinetics of an immediate early gene. J Bone Min Res.

[32] Kanzawa M, Sugimoto T, Kanatani M, Chihara K (2000). Involvement of osteoprotegerin/osteoclastogenesis inhibitory factor in the stimulation of osteoclast formation by parathyroid hormone in mouse bone cells. Eur J Endocrinol.

[33] Al-Omari FA, Kuroshima S, Uto Y, Uchida Y, Sawase T. Effect of intraoral administration of parathyroid hormone on osseous and soft tissue healing around implants in ovariectomized rat maxillae. Clin Oral Implants Res. 2023. 10.1111/clr.14227 38124678

[34] Steffi C, Shi Z, Kong CH, Wang W (2018). Modulation of Osteoclast interactions with Orthopaedic Biomaterials. J Funct Biomater.

[35] Chen S, Guo Y, Liu R, Wu S, Fang J, Huang B (2018). Tuning surface properties of bone biomaterials to manipulate osteoblastic cell adhesion and the signaling pathways for the enhancement of early osseointegration. Colloids Surf B Biointerfaces.

[36] O’Brien CA, Morello R (2018). Modeling rare bone diseases in animals. Curr Osteoporos Rep.

